# Fat beyond muscle: Assessing epimuscular fat of the lumbar spine and its association with vertebral level, demographics, BMI, and low back pain

**DOI:** 10.1016/j.bas.2024.103916

**Published:** 2024-10-16

**Authors:** Jacopo A. Vitale, Anne F. Mannion, Daniel Haschtmann, Mario Ropelato, Tamás F. Fekete, Frank S. Kleinstück, Markus Loibl, Tina Haltiner, Fabio Galbusera

**Affiliations:** a Spine Group, Schulthess Klinik, Zürich, Switzerland; b Zürich University of Applied Sciences ZHAW, Zürich, Switzerland

**Keywords:** Muscle, Fat infiltration, Cross-sectional area, LBP, COMI

## Abstract

**Introduction:**

Epimuscular fat (EF) has rarely been studied in the context of low back pain (LBP).

**Research question:**

This study aims to assess the presence and extent of EF in the lumbar muscles and its association with vertebral level in patients with low back disorders and to explore correlations between EF, demographics, BMI, and LBP.

**Material and methods:**

T2 axial MRIs from L1 to L5 were manually segmented to analyze the cross-sectional area (CSA) of EF (mm^2^), and fat infiltration (FI,%) of 40 patients (23 females, 17 males; mean age:65.9 years) with lumbar degenerative pathologies awaiting a surgical procedure. COMI, LBP, demographic, and clinical data were extracted from the institutional registry. Statistical analyses included Wilcoxon and Mann-Whitney tests for differences in EF between sides and sexes, the Friedman test for EF size differences among lumbar levels, and Spearman’s correlation for associations, adjusted for BMI, age, and sex.

**Results:**

EF was found in 77.5% of subjects at L1, 92.5% at L2, 100% at L3 and L4, and 95.0% at L5. EF was significantly larger at L4 (253.1 ± 183.6 mm^2^) and L5 (220.2 ± 194.9 mm^2^) than at L1 (36.1 ± 37.8 mm^2^) and L2 (72.2 ± 84.4 mm^2^). No significant EF differences were found between sides and sexes. EF correlated strongly with BMI (r_s_ = 0.65,p < 0.001) and moderately with FI (r_s_ = 0.31,p = 0.04), though its correlation with FI was not significant after adjustment. EF did not correlate with COMI scores but correlated with LBP in the adjusted analysis (r_s_:0.31,p = 0.04).

**Discussion and conclusion:**

EF is present across all lumbar levels, with higher concentrations at L4 and L5, and a significant correlation between EF and LBP intensity was observed. The present findings are limited to a specific subset of patients with lumbar degenerative disorders who are awaiting surgical procedures.

## Highlights

• Epimuscular fat (EF) has rarely been studied in the context of low back pain (LBP).
• EF is present across all lumbar levels, with higher concentrations at L4 and L5.
• Significant positive correlations were observed between EF and BMI, and moderate correlations between EF and age.
• EF showed a significant correlation with LBP intensity scores.

## 1 Introduction

Low back pain (LBP) is a common condition affecting a significant portion of the global population, leading to substantial morbidity and economic burden (Buchbinder et al., 2018). It is estimated that up to 80% of individuals will experience LBP at some point in their lives, making it one of the most common musculoskeletal complaints (Andersson, 1999). Chronic LBP is a multifactorial condition with numerous potential causes including degenerative conditions (osteoarthritis, degenerative disc herniation, spinal stenosis, spondylolisthesis), infection, inflammatory conditions, fractures, and tumours, with lifestyle (obesity, lack of physical activity) and psychosocial factors (anxiety and depression, work dissatisfaction) playing a major role (Knezevic et al., 2021). Chronic LBP significantly impacts quality of life and functional ability, often leading to disability and prolonged periods of work absenteeism (Husky et al., 2018). Among the various factors associated with LBP, the structural and compositional properties of the paraspinal muscles have garnered considerable attention (Ranger et al., 2017; Noonan and Brown, 2021). The cross-sectional area (CSA) and fat infiltration (FI), or myosteatosis, of these muscles are key parameters that have been examined to understand their role in LBP (Berry et al., 2020). Research has shown that a decrease in CSA due to muscle atrophy can lead to reduced support for the spine and increased mechanical strain on spinal structures, contributing to the development and persistence of LBP (Wan et al., 2015; Seyedhoseinpoor et al., 2022). In addition to muscle atrophy, FI within the paraspinal muscles is another significant factor associated with LBP. FI involves the replacement of muscle fibers with fat, which can impair muscle function and reduce the efficiency of force generation and transmission (Mengiardi et al., 2006; Hamrick et al., 2016). This infiltration is often quantified as a percentage of fat within the muscle cross-section, and higher levels of FI have been correlated with greater LBP intensity and disability (Kjaer et al., 2005), although the results are not entirely conclusive (Banitalebi et al., 2022).

Epimuscular fat (EF), defined as the fat content located outside the epimyseal border between the multifidus and erector spinae muscles and the thoracolumbar fascia, represents an additional factor that may influence LBP (Berry et al., 2018). Unlike FI, which directly infiltrates muscle fibers, EF is situated externally and may impact muscle biomechanics, force generation, and intermuscular connectivity (Finni et al., 2023). The exact directionality of the relationship between EF and LBP is unclear: EF might either impair muscle mechanics, contributing to the development or persistence of LBP, or represent an adaptive response to muscle dysfunction caused by LBP. Nevertheless, the specific EF-LBP relationship, and its distribution across different spinal levels, remains underexplored. To the best of our knowledge, to date, only one study has examined EF in participants with and without LBP (Rosenstein et al., 2024). The authors reported that EF was more frequently observed at the L4-S1 levels in participants with LBP compared to controls and that quantitative measures of EF were significantly correlated with BMI, age, and LBP status. However, no correlations were found between epimuscular fat and the duration or intensity of LBP.

While reduced CSA and increased FI have been widely studied in the context of LBP, the role of EF remains inadequately explored. Given that EF is anatomically positioned to potentially affect muscle mechanics (Finni et al., 2023), its contribution to LBP warrants thorough investigation. Therefore, this study aims to assess the presence and extent of EF in the lumbar spine and its association with vertebral level (L1-L5) in patients with low back disorders. Furthermore, it seeks to investigate the potential correlations between EF and patient demographics, body mass index (BMI), and LBP intensity. We hypothesize that: 1) EF will be present to varying extents across the lumbar spine, with higher areas at L4-L5; 2) EF will be positively correlated with higher BMI and older age; and 3) higher levels of EF will be associated with increased LBP intensity.

## 2 Methods

### 2.1 Study design and subjects

This retrospective cross-sectional study followed the STROBE guidelines for reporting (von Elm et al., 2007). Data from 40 consecutive patients with low back disorders who had been seen by the Unit of Spine Surgery, Orthopaedics, and Neurosurgery at the Schulthess Klinik (Zürich, Switzerland) between 2018 and 2019 were extracted and analyzed. All patients who had undergone lumbosacral MRI for clinical purposes at baseline, had any lumbar degenerative pathology (e.g., stenosis, disc herniation, spondylolisthesis) as observed in the MRIs and reported by radiologists in the medical records, and awaiting surgical treatment, were included in the study. Patients were excluded if they had a history of spinal surgery or vertebral fracture, major lumbar spine deformities/abnormalities, incorrect anatomical level of MRIs (i.e., not between L1–L5), or MRIs with excessive noise or a small field of view not including all muscles of interest.

Patient-reported outcome measures (i.e., the “Core Outcome Measures Index” [COMI] (Mannion et al., 2009) and Low Back Pain intensity [LBP]) and demographic and clinical data (e.g., disease, age, sex, or BMI) were extracted from the institutional registry of all patients undergoing surgical treatment for spine disorders. Data use from the registry was approved by the Kantonalen Ethikkommission Zürich (BASEC-Nr: 2023-01683). All patients included in this study provided written consent for coded data use for research purposes, which the Ethics Committee accepted as informed consent for the present study. The research project was conducted under the Declaration of Helsinki, the principles of Good Clinical Practice, the Human Research Act (HRA), and the Human Research Ordinance (HRO).

### 2.2 MRI-based muscles and epimuscular fat segmentation

**Fig. 1:**
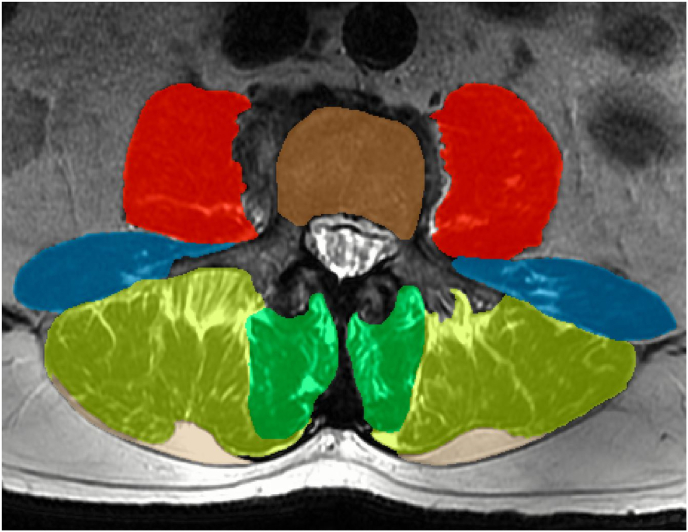
Example of segmentation of paravertebral muscles and epimuscular fat at L3 level using ITK-SNAP software. Red: psoas major; blue: quadratus lumborum; yellow: erector spinae; green: multifidus; brown: vertebral body area; beige: epimuscular fat. (For interpretation of the references to colour in this figure legend, the reader is referred to the Web version of this article.)

Axial T2-weighted MRIs of the lumbar spine were downloaded from our Institutional image database and manually segmented to determine lumbar spine muscle and fat characteristics. One single image for each lumbar level (i.e. the one closest to the centroid of the vertebral body) was segmented for a total of 5 images for each patient (L1 – L5). In detail, for each image, one investigator manually segmented erector spinae, multifidus, quadratus lumborum, psoas major (all muscles bilaterally), total vertebral body, and, when present, EF, for the right and left side. EF was defined as the fat content outside of the epimyseal border, between the multifidus and erector spinae muscles and the thoracolumbar fascia (Fig. 1). A second investigator performed all segmentations for a subset of 10 subjects to assess inter-rater reliability for primary outcomes. Manual segmentation was performed using the ITK-SNAP free software (PA, USA), following previous recommendations for the manual segmentation of paraspinal muscles from axial MRIs (Anstruther et al., 2023; Crawford et al., 2017). The outcome measures from MRIs were:

1) Quantitative EF surrounding the erector spinae and multifidus muscles, expressed in mm^2^, for each lumbar level, bilaterally.
2) Total quantitative EF, expressed in mm^2^, calculated by summing 10 quantitative measurements: two sides x five levels.
3) CSA, expressed in mm^2^, for the multifidus and erector spinae muscles, for each lumbar level, bilaterally, excluding EF.
4) FI within the muscles, expressed in %, for the multifidus and erector spinae muscles, for each lumbar level, bilaterally. Specifically, as previously presented (Niemeyer et al., 2022; Vitale et al., 2024), the fat fraction was calculated by classifying each pixel as either fat or lean tissue using a basic Otsu binary thresholding method, implemented through the scikit-image Python library for image processing.
5) Vertebral body area (VBA), expressed in mm^2^, for each lumbar level. Quantitative EF measures were also normalized for VBA of L4 (see “*Statistical Analyses*” paragraph for details).

### 2.3 Epimuscular fat qualitative score

As recently presented by Rosenstein et al. (2024), we assigned a qualitative rating for each lumbar level and side, assessing the presence and extent of EF along the border of the multifidus and erector spinae muscles. The ratings consisted of a 5-point scale, with 0 indicating no presence of EF, and 5 indicating the presence of EF along the full length of the border of the muscles. Lastly, a total qualitative rating score was calculated by summing all the EF ratings for each level and side (a total of 10).

### 2.4 COMI and LBP

Patients' COMI and LBP scores, collected at baseline (i.e. the day before surgery), were extracted from our institutional registry. The COMI consists of seven items that assess five domains: pain, function, symptom-specific well-being, quality of life, and disability. The COMI summary score is the average of all five domain scores, and it ranges between 0 (best status) and 10 (the worst possible status) (Mannion et al., 2009). LBP intensity in the last week is rated, within the COMI questionnaire, on a 0–10 numerical rating scale. Both COMI summary and LBP scores were used as outcome measures.

### 2.5 Statistical analysis

One investigator manually performed all segmentations on MRI images for all subjects and a second investigator performed all segmentations for a subset of 10 subjects. To assess inter-rater reliability, the Intraclass Correlation Coefficient (ICC) estimates and their 95% confidence intervals (CIs) were calculated based on a single rating, consistency, 2-way mixed-effects model. As previously described (Koo and Li, 2016), values less than 0.5 were considered indicative of poor reliability, values between 0.5 and 0.75 of moderate reliability, values between 0.75 and 0.9 of good reliability, and values greater than 0.90 of excellent reliability.

Descriptive statistics (mean or median ± SD and 95% confidence interval [95% CI], when relevant) for all outcome measures were calculated. The normality distribution of all variables was evaluated and checked with the Shapiro–Wilk test. Given a nonnormal distribution for all parameters, the nonparametric Wilcoxon test was performed to detect possible differences in quantitative EF, expressed in mm^2^, between the right and left side, for each level, and the Mann-Whitney test was used to assess differences between female and male subjects. Chi-squared tests were used to evaluate possible differences between observed data and expected data of qualitative EF ratings between the right vs left side and between sexes. The nonparametric Friedman test followed by Dunnʼs multiple comparisons was used to compare the amount of EF and the qualitative EF ratings among different lumbar levels, from L1 to L5, for each side. Effect size for significant pairwise comparisons was calculated using Cohen’s d and considered to be either trivial (effect size:<0.20), small (0.21–0.60), moderate (0.61–1.20), large (1.21–2.00), or very large (>2.00) (Hopkins et al., 2009).

The existence of a correlation between total EF (quantitative and qualitative) and FI, age, BMI, COMI, and LBP was tested by the means of the Spearman correlation index (r_s_). In addition, adjusted partial correlations were conducted for quantitative and qualitative EF measures, accounting for participants' BMI, age, and sex. Spearman’s correlation coefficients were interpreted using the following correlation guidelines: r < 0.3 as small/weak, 0.3 < r < 0.5 as moderate, and 0.5 < r < 1.0 as large (Mukaka, 2012). Correlations were considered significant when r > 0.3 and p < 0.05. All the analyses involving quantitative EF were also performed with EF measures normalized for the total vertebral body of L4. All analyses with normalized EF were in line with the absolute EF measures.

The level of significance was set at p < 0.05. Statistical analysis was performed using GraphPad Prism version 9.5.1 for Windows (GraphPad Software, San Diego, CA, USA) and the open-source Pingouin package in Python 3.9 (Vallat, 2018).

## 3 Results

Overall, there was good to excellent inter-rater reliability (ICCs >0.75) for all CSA and FI measures, and excellent reliability (ICCs >0.90) for EF measures.

### 3.1 Participants

The sample was composed of n = 40 adult patients (n = 23 females, n = 17 males; mean age: 65.9 ± 13.9 years old; mean BMI: 27.4 ± 4.6) with back disorders, awaiting surgical treatment. The most represented pathologies were disc herniation (n = 13, 32.5%), central stenosis (n = 9, 22.5%), and spondylolisthesis (n = 8, 20.0%). Participants' characteristics are presented in Table 1.

**Table 1:** Baseline characteristics of the patients.

Variable	Descriptive statistics	Value	
**Female gender**	*(n, %)*	n = 23, 57.5%	

**Age***(years)*	Mean ± SD	65.9 ± 13.9	
	Range (min – max)	28.6–87.2	
	95% CI (lower – upper)	61.5–70.3	
**Height***(cm)*	Mean ± SD	167.6 ± 8.2	
	Range (min – max)	153.0–190.0	
	95% CI (lower – upper)	165.0–170.2	
**Weight***(kg)*	Mean ± SD	77.1 ± 15.0	
	Range (min – max)	50.0–116.0	
	95% CI (lower – upper)	72.2–81.9	
**BMI***(kg/m*^*2*^*)*	Mean ± SD	27.4 ± 4.6	
	Range (min – max)	18.9–36.6	
	95% CI (lower – upper)	25.9–28.9	
**CSA***(mm*^*2*^*)*		**Multifidus**	**Erector Spinae**
	Mean ± SD	730.7 ± 351.9	1773 ± 547
	Range (min – max)	73.6–2514	333.8–3698
	95% CI (lower – upper)	696.1–765.4	1719–1826
**FI***(%)*		**Multifidus**	**Erector Spinae**
	Mean ± SD	31.4 ± 10.9	22.2 ± 13.7
	Range (min – max)	6.8–61.7	2.7–78.9
	95% CI (lower – upper)	30.3–32.5	20.8–23.6
**Baseline COMI***(score)*	Mean ± SD	7.5 ± 1.5	
	Range (min – max)	4.4–10	
	95% CI (lower – upper)	7.0–8.0	
**LBP***(scores)*	Mean ± SD	5.5 ± 2.6	
	Range (min – max)	0–10	
	95% CI (lower – upper)	4.6–6.2	
**Main pathology***(n, %)*	Central stenosis	9, 22.5%	
	Disc degeneration	3, 7.5%	
	Disc herniation	13, 32.5%	
	Facet joints arthrosis	1, 2.5%	
	Foraminal stenosis	1, 2.5%	
	Lateral stenosis	5, 12.5%	
	Spondylolisthesis	8, 20.0%	

Values are presented as means ± standard deviations, range (minimum-maximum), and 95% confidence interval. *Abbreviations*: BMI, Body Mass Index; CSA, cross-sectional area; FI, fat infiltration; COMI, Core Outcome Measures Index.

### 3.2 Side, level, and sex differences

The presence of EF was detected in n = 31 subjects (77.5%) at L1, n = 37 (92.5%) at L2, n = 40 (100%) at L3 and L4, and n = 38 (95.0%) at L5. No difference was detected in the amount of EF, expressed in mm^2^, between the right and left side (Table 2) and between sexes (Table 3), at each lumbar level, except L5 level that showed a higher EF on the right vs the left side (delta difference: 22.5 mm^2^, p = 0.008, ES: 0.11, trivial). In addition, the Chi-Square tests also highlighted no differences between sides and sexes in the qualitative EF ratings at each lumbar level (p > 0.05).

**Table 2:** Amount of epimuscular fat on the right and left side at each lumbar level (L1-L5).

	Right EF *(mm*^*2*^)	Left EF *(mm*^*2*^*)*	Significance and ES
**L1** (n = 31)	34.4 ± 42.3	41.0 ± 38.8	p = 0.16
**L2** (n = 37)	73.0 ± 81.2	73.9 ± 95.2	p = 0.85
**L3** (n = 40)	150.4 ± 142.1	153.6 ± 132.7	p = 0.70
**L4** (n = 40)	263.3 ± 197.7	242.1 ± 180.9	p = 0.34
**L5** (n = 38)	235.8 ± 215.3	213.3 ± 183.2	p = 0.008, ES: 0.11, *trivial*
**SUM**	757.6 ± 99.4	723.7 ± 86.7	p = 0.34

Values are presented as means ± standard deviations. *Abbreviations*: EF, epimuscular fat; ns, no statistical difference; ES: Effect Size.

**Table 3:** Gender differences in epimuscular fat at each lumbar level (L1-L5), age and BMI.

	Females EF *(n = 23*)	Males EF *(n = 17)*	Significance
**L1**	40.3 ± 46.4	29.9 ± 22.7	p = 0.83
**L2**	82.5 ± 105.1	59.4 ± 51.7	p = 0.57
**L3**	154.2 ± 140.1	148.8 ± 128.6	p = 0.96
**L4**	232.5 ± 184.5	281.0 ± 188.3	p = 0.41
**L5**	197.7 ± 169.4	251.2 ± 232.5	p = 0.53

**Average**	147.9 ± 122.7	155.0 ± 103.4	p = 0.78
**Sum**	679.5 ± 572.4	750.3 ± 528.2	p = 0.36

**Age***(years)*	67.7 ± 13.3	63.9 ± 14.9	p = 0.50

**BMI***(kg/m*^*2*^*)*	26.4 ± 4.6	28.6 ± 4.6	p = 0.14

Values are presented as means ± standard deviations. *Abbreviations*: EF, epimuscular fat; ns, no statistical difference; BMI, Body Mass Index.

**Fig. 2:**
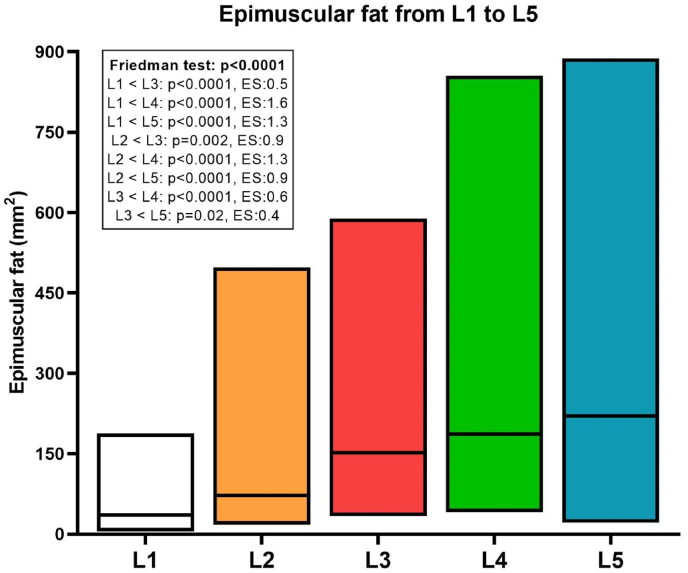
Floating bars, with median (central line), minimum and maximum values (lower and upper lines) of epimuscular fat at L1 (white bar), L2 (orange bar), L3 (red bar), L4 (green bar), and L5 (blue bar). In the box, details of Dunn’s multiple comparisons and effect size (ES) are reported. (For interpretation of the references to colour in this figure legend, the reader is referred to the Web version of this article.)

**Fig. 3:**
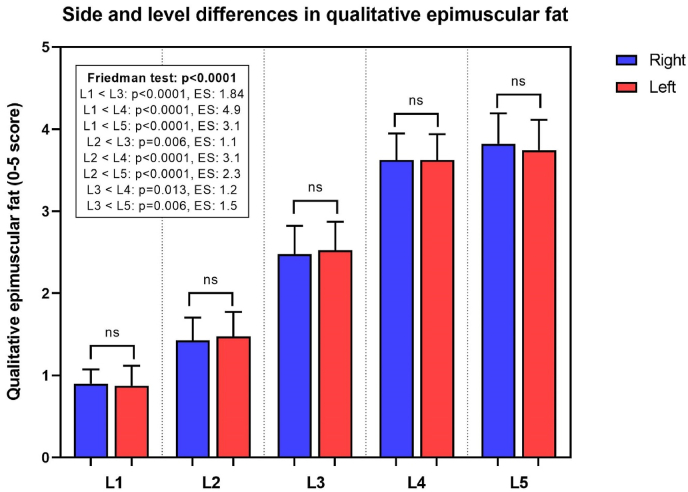
Histograms with mean and 95% CI of qualitative epimuscular fat ratings (from 0 to 5) for the right (blue) and left (red) side, at each lumbar level (L1 – L5). In the box, details of Dunn’s multiple comparisons and effect size (ES) are reported. (For interpretation of the references to colour in this figure legend, the reader is referred to the Web version of this article.)

EF significantly differed among lumbar levels (p < 0.0001) with higher amounts of fat displayed at L4 and L5 levels. In detail, the extent of EF was greater at L5 (220.2 mm^2^) and L4 (253.1 mm^2^) compared with L1 (36.1 mm^2^) and L2 (72.2 mm^2^), with p < 0.0001 for each comparison. Fig. 2 reports graphically the inter-level differences in the amount of EF, expressed in mm^2^ and averaged across both the right and left sides whereas Fig. 3 shows side and inter-level differences in qualitative EF scores.

### 3.3 Correlations with BMI, FI, age, and LBP

**Fig. 4:**
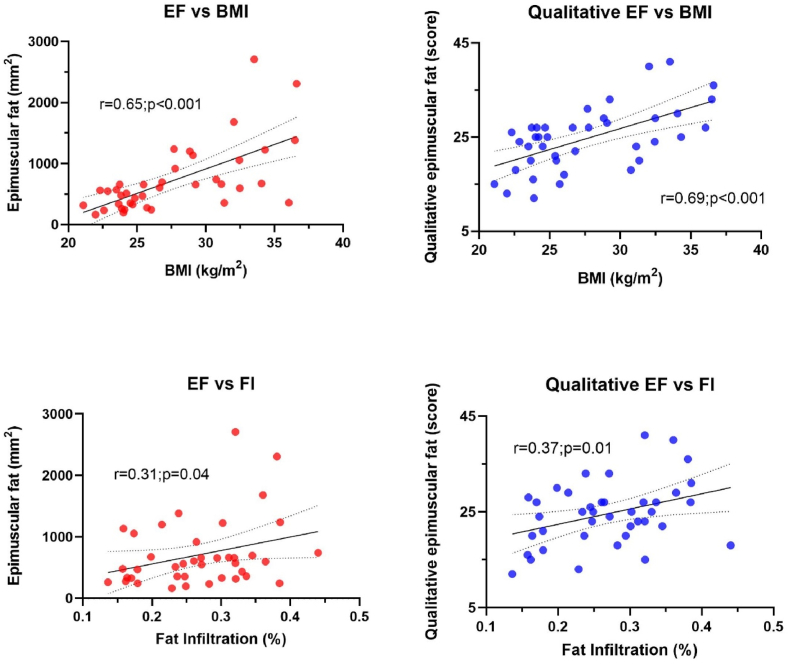
Crude correlations between total quantitative epimuscular fat (left panels, red circles) or qualitative epimuscular fat (right panels, blue circles) vs Body Mass Index (BMI) and Fat Infiltration (FI). (For interpretation of the references to colour in this figure legend, the reader is referred to the Web version of this article.)

Table 4 shows crude and adjusted partial correlations between total quantitative and qualitative EF measures vs fat infiltration, age, BMI, COMI, and LBP. Fig. 4 graphically shows the crude correlations between total quantitative and qualitative EF vs BMI and FI. Large positive correlations, both crude and adjusted, between total EF and BMI, were detected (quantitative: r_s_:0.65, p < 0.001; qualitative: r_s_:0.69, p < 0.001) whereas moderate crude correlations were observed between total EF and FI (quantitative: r_s_:0.31, p = 0.04; qualitative: r_s_:0.37, p = 0.01) but these correlations did not remain significant in the adjusted analysis. Age showed a moderate crude correlation only with qualitative EF scores (r_s_:0.33, p = 0.03), but not in the adjusted analysis. COMI scores did not correlate significantly with qualitative or quantitative EF measures whereas LBP, in, the adjusted analysis, correlated significantly with total quantitative EF (r_s_: 0.31, p = 0.04). None of these relationships were significant when using the leg pain or maximum pain scores extracted from COMI.

**Table 4:** Crude and adjusted partial correlations between total quantitative and qualitative epimuscular fat scores vs Fat Infiltration, age, BMI, COMI, and LBP.

	Crude correlation		Partial Adjusted correlation *(for age, sex, and BMI)*	
**Total quantitative EF *(mm***^***2***^***)***	**r**_**s**_	**p-value**	**r**_**s**_	**p-value**

Fat infiltration *(%)*	**0.31**	**0.04**	0.08	0.62
Age *(years)*^a^	0.24	0.13	0.20	0.21
BMI *(kg/m*^*2*^*)*^a^	**0.65**	**<0.001**	**0.71**	**<0.001**
COMI *(score)*	0.09	0.55	0.26	0.10
LBP *(score)*	0.14	0.39	**0.31**	**0.04**

**Total qualitative EF *(score)***	**r**_**s**_	**p-value**	**r**_**s**_	**p-value**

Fat infiltration *(%)*	**0.37**	**0.01**	0.03	0.84
Age *(years)*^a^	**0.33**	**0.03**	0.29	0.07
BMI *(kg/m*^*2*^*)*^a^	**0.69**	**<0.001**	**0.64**	**<0.001**
COMI *(score)*	0.07	0.66	0.19	0.25
LBP *(score)*	0.11	0.47	0.24	0.14

**Fat infiltration *(%)***	**r**_**s**_	**p-value**	**r**_**s**_	**p-value**

Age *(years)*^a^	**0.49**	**0.002**	**0.55**	**<0.001**
BMI *(kg/m*^*2*^*)*^a^	0.26	0.11	**0.37**	**0.02**
COMI *(score)*	0.17	0.28	0.30	0.07
LBP *(score)*	<0.01	0.97	0.08	0.63

Spearman’s rho (r_s_) used for all correlations. Abbreviations: EF, epimuscular fat; ns, no statistical difference; BMI, Body Mass Index; COMI, Core Outcome Measure Index; LBP, Low Back Pain.

a Age was adjusted only for BMI and sex and BMI was adjusted only for age and sex.

A positive correlation between FI and age was detected in both the crude (r_s_ = 0.49, p = 0.002) and adjusted analyses (r_s_ = 0.55, p < 0.001). A moderate correlation was observed between FI and BMI (r_s_ = 0.37, p = 0.02) only when adjusted for age and sex. No significant correlations were detected between FI and COMI or LBP. Table 4 displays the crude and adjusted partial correlations between FI vs age, BMI, COMI, and LBP.

## 4 Discussion

To our knowledge, this is the first study to investigate the presence and extent of lumbar muscle EF and its association with vertebral level, demographics, BMI, and LBP in patients with low back disorders. The primary findings indicated that EF was present at all lumbar levels, with higher amounts at L4 and L5. Significant positive correlations were observed between EF and BMI, and moderate correlations between qualitative EF scores and age. While EF correlated with FI in crude analyses, these correlations were not significant after adjustments. Importantly, LBP scores showed a significant, albeit moderate, correlation with quantitative EF in the adjusted analysis. While some of the correlations align with expectations based on known associations between adiposity, BMI, and aging, the novel aspect of our study is its focus on EF, rather than FI, and the relationship between EF and LBP. Our initial hypotheses were confirmed.

The results of this study align with the existing literature on the structural and compositional properties of paraspinal muscles concerning LBP. This study complements these findings by focusing on EF, an external fat layer, demonstrating that it too is significantly correlated with LBP intensity scores. One of the strengths of this study is that it comprehensively analyzed EF across multiple lumbar levels, including both quantitative and qualitative measures of EF. Additionally, there was a significant positive correlation between the qualitative and quantitative EF measures (r_s_: 0.76, p < 0.0001) suggesting that both methods likely capture the same underlying construct. All analyses involving quantitative EF were also performed with EF measures normalized for the total vertebral body area of L4, and the results were consistent with those using absolute EF measures. Lastly, the manual segmentation method used demonstrated excellent inter-rater reliability, ensuring the robustness of the findings.

We observed the presence of EF in all lumbar levels, particularly at L4 and L5. Although our study did not include a control group, this observation is consistent with the findings reported by Rosenstein et al. (2024), which indicated that EF at the L4-L5 and L5-S1 levels was more frequently observed in participants with LBP than in controls. This result is also in line with previous studies showing that FI within muscles is often more pronounced at lower lumbar levels (Kjaer et al., 2005) or that greater changes in paraspinal muscle morphology over time (e.g. atrophy or FI) occur at L5-S1 compared to L3-L4 level (Fortin et al., 2014). This observation aligns with the fact that the L5-S1 is subjected to the highest mechanical load within the lumbar spine (Ignasiak et al., 2018), resulting in greater stress at that level (Fortin et al., 2014).

Data on the relationship between EF and BMI, age, and sex are limited. We observed a strong positive correlation between EF (both quantitative and qualitative) and BMI, both in the crude analysis and after adjusting for age and sex. This finding aligns with a previous study by Rosenstein et al. (2024), which linked higher BMI to increased EF in LBP patients. However, most previous studies have focused on the association between FI and BMI. These studies have yielded inconsistent results, with most of them showing no significant correlation between BMI and total FI in patients with LBP or back disorders (Vitale et al., 2024; Hildebrandt et al., 2017). In line with this statement, we observed no significant crude correlation between FI and BMI. However, the analysis adjusted for the possible confounders of age and sex showed a significant association between FI and BMI. Therefore, it can be stated that increased BMI does not necessarily translate to increased FI in the lumbar spine muscles, but it appears that high BMI is a significant risk factor for increased EF deposition. While it is well-established that higher BMI is associated with LBP in general populations (Nitecki et al., 2023; Hershkovich et al., 2013), we did not find a direct correlation between BMI and LBP in our sample of patients with specific lumbar disorders (r_s_:0.25, p = 0.11). This discrepancy may be due to the nature of our patient cohort, which included individuals with known spinal pathologies, where other factors such as EF may play a more critical role. Our results suggest that increased EF, which is strongly correlated with BMI, could act as an intermediary between BMI and LBP. In particular, the association between EF and LBP persisted even after adjusting for BMI, highlighting EF as a potential key contributor to LBP rather than BMI directly.

Regarding age, we observed a moderate crude correlation with qualitative ratings of EF, suggesting that as patients age, there may be a tendency for increased deposition of EF. This result is partially in line with the findings by Rosenstein et al. (2024), who highlighted a moderate correlation between age and quantitative EF measures, but not with qualitative EF scores. Previous studies have predominantly focused on the impact of aging on CSA and FI in patients with LBP. Goodpaster et al. (2001) demonstrated that aging is associated with an increase in FI and a decrease in CSA and Dallaway et al. (2020) observed that older subjects had higher FI values in the erector spinae and multifidus muscles than younger individuals. Additionally, Vitale et al. (2024) reported a significant increase in FI for each lumbar spine muscle with advancing age in patients with back disorders. In the present study, we also observed strong crude and adjusted correlations between patients' age and FI, supporting the notion that aging contributes to increased FI in lumbar spine muscles more significantly than it does to EF. In addition, we did not detect any sex difference in the presence or content of EF. This finding is partially in line with the study by Rosenstein et al. (2024), which showed only a weak correlation between sex and qualitative EF measures. However, it contrasts with some of the literature on general fat distribution, which often shows sex-specific patterns, particularly in subcutaneous fat and FI. For example, Crawford et al. (2017) found that healthy male subjects had lower lumbar muscle FI than healthy females. Similarly, Vitale et al. observed higher FI and lower CSA in the multifidus, erector spinae, quadratus lumborum, and psoas major muscles in females compared with males. Further investigations are needed to better understand possible sex differences in EF content.

COMI scores did not show a significant correlation with EF measures. Nevertheless, we observed a significant correlation between quantitative EF measures and LBP intensity scores, but only in the analysis that was adjusted for the possible confounders of age, sex, and BMI. Our results are partially consistent with previous findings that showed that quantitative and qualitative EF measures correlated with LBP status (i.e., being an LBP patient or healthy control) but not with LBP duration and intensity (Rosenstein et al., 2024). While the relationship between EF and pain is largely unstudied and warrants further investigation, there are various mechanisms that could explain our findings. Increased EF can lead to local inflammation, which can sensitize nerve endings and amplify pain perception (Risbud and Shapiro, 2014), or to altered biomechanics and reduced efficiency of the paraspinal muscles, potentially contributing to mechanical back pain (Finni et al., 2023). While our study supports the hypothesis that EF is associated with LBP, it is important to consider alternative explanations for this correlation. There may be a reverse causality where higher LBP leads to reduced mobility, which in turn could contribute to an increase in BMI and subsequent EF deposition (Airaksinen et al., 2006). It is also worth noting that the hypothesis of EF leading to local inflammation may be more applicable to patients with non-specific LBP (Seyedhoseinpoor et al., 2022; Kalichman et al., 2017). In contrast, the patients in our study had specific lumbar disorders with a known cause of pain, which may suggest different mechanisms of pain and fat deposition. Future research should aim to disentangle the potential pathways by considering patient mobility, BMI changes, and the role of inflammation in both specific and non-specific LBP populations.

It is important to highlight that the association between LBP and FI has been studied more often than that between LBP and EF, but the results on this association are not unequivocal. Some studies have identified a clear association between increased FI in paraspinal muscles and the presence and/or severity of LBP. For instance, Kjaer et al. (2005) found that individuals with LBP had significantly higher levels of FI in the multifidus muscles compared with healthy controls, and Fortin et al. (Fortin and Macedo, 2013) reported that higher degrees of FI were associated with greater LBP intensity and disability. However, other cross-sectional studies do not corroborate these findings. Mengiardi et al. (2006) and Ploumis et al. (2011) reported no association between LBP intensity and paraspinal muscle FI or size. Furthermore, Fortin et al. (2014) did not observe any significant association between LBP frequency or intensity and changes in CSA and FI. Noteworthy is that in the present study, we did not detect any significant correlation between FI and LBP or COMI scores. This highlights the potential relevance of considering EF when studying the relationship between LBP and lumbar muscle and fat characteristics in future studies.

We must acknowledge some limitations of the study. First, the study’s relatively small sample size (n = 40), its heterogeneity (i.e. patients with different lumbar pathologies), and the lack of a healthy control group with no LBP may limit the generalizability of the findings. Second, the different pathologies in our sample could lead to higher baseline LBP scores, potentially confounding the analyses. Additionally, it is also important to note that most other studies have looked at non-specific chronic LBP, whereas our patients had specific spinal disorders with a supposedly known cause of their pain and this difference in patient population might impact the comparability of our findings. Third, the retrospective cross-sectional design precludes causal inferences about the relationship between EF and LBP. Fourth, although the use of T2-weighted MRI in this study provides a robust imaging contrast that differentiates between muscle and fat tissues, it may not adequately detect or differentiate between fat and inflammatory changes within the muscles. More advanced techniques like Dixon MRI or MR spectroscopy may offer superior resolution and provide detailed fat composition analysis. Fifth, the correlation analysis was also performed using the leg pain or maximum pain scores extracted from COMI, but no significant association with EF was observed, suggesting that EF may be more specifically associated with LBP intensity rather than other pain dimensions.

## 5 Conclusion

EF is present across all lumbar levels, with higher concentrations at L4 and L5. Significant positive correlations were observed between EF and BMI, and moderate correlations between EF and age. EF showed a significant correlation with LBP intensity scores in adjusted analyses, suggesting that EF may be a relevant factor in the presentation of LBP. Our findings also suggest that high BMI, while associated with increased EF, may not directly translate into LBP but rather contributes to EF accumulation, which in turn may exacerbate pain. However, given the retrospective cross-sectional design of the study, future research should focus on longitudinal designs, use advanced imaging techniques, involve fat biopsies, and include larger, more homogeneous samples with healthy controls to establish causality and further elucidate the role of EF in LBP.

## Funding

This research did not receive any specific grant from funding agencies in the public, commercial, or not-for-profit sectors.

## Declaration of competing interest

The authors declare that they have no known competing financial interests or personal relationships that could have appeared to influence the work reported in this paper.
